# Effects of Self-Regulated Learning on Student’s Reading Literacy: Evidence From Shanghai

**DOI:** 10.3389/fpsyg.2020.555849

**Published:** 2021-01-13

**Authors:** Xiang Qi

**Affiliations:** Department of Public Administration, Business School, University of Shanghai for Science and Technology, Shanghai, China

**Keywords:** self-regulated learning, cognitive strategy, metacognition, enjoyment, literacy

## Abstract

Many empirical studies have been conducted to investigate self-regulated learning (SRL) in the Western countries. Less well investigated is the SRL in the Chinese Mainland students and how it affects their academic achievement. On the basis of PISA 2009, this paper is aimed at exploring the SRL of 15-year-old Shanghai students, as measured by cognitive strategy (elaboration and memorization), metacognition (metacognition in understanding and remembering, metacognition in summarizing, and control strategy), and motivational belief (enjoyment of reading). In the aspect of SRL nature, the results reveal that 15-year-old students in Shanghai use elaboration strategy frequently and seldom use memorization strategy, and that they have high metacognition in understanding, remembering, and summarizing but have low control strategy, and that their enjoyment of reading is relatively high. In the aspect of SRL’s consequence for reading literacy, findings from multilevel linear regression corroborate previous evidence from the Western countries about the effect of SRL on academic achievement. Specifically, elaboration strategy, metacognition in understanding and remembering, metacognition in summarizing, control strategy, and enjoyment of reading are conducive to students’ reading literacy, while memorization strategy exerts a significantly negative effect on reading literacy. The findings could be useful in helping us to gain a better understanding of Shanghai students’ SRL.

## Introduction

Self-regulated learning (SRL) in education has received increasing attention over the last three decades. This growing interest can be explained from two different perspectives. In an era of rapid development of science and technology, there is a growing emphasis on the acquisition of SRL to facilitate lifelong learning (Clark, 2012). On the other hand, SRL is viewed as a means to foster and develop “critical thinkers.” In this line of thinking, researchers from diverse theoretical backgrounds have investigated students’ self-initiated efforts to learn and self-instruction as instances of SRL since the mid-1980s (Zimmerman and Kitsantas, 2014). SRL refers to a self-directive learning process where learners actively employ metacognitive, motivational, and behavioral strategies (Zimmerman, 1986). These strategies are invoked during different phases of the learning process, for example, to activate self-motivational belief in forethought phase (Zimmerman, 2002).

Theoretically, SRL takes a broad perspective on student learning than other approaches to learning because it entails not only cognition but also motivation and affect, as well as social context (Pintrich, 2000). Students with higher SRL could have strong motivation and use adaptive cognitive strategy, and as a consequence, they are more likely to succeed academically (Zimmerman, 2002). Many empirical studies have been conducted to investigate the extent to which students perform SRL as well as the effect of SRL on students’ academic achievement in Western countries (e.g., Nota et al., 2004; León et al., 2015). Some Chinese scholars take the premise, that the effectiveness of SRL in Western counties is also applicable in China, for granted, so they shift their research focus to SRL’s determinants (e.g., Li and Zheng, 2018; Guo and Wei, 2019; Sun and Tang, 2019; Wang et al., 2019; Guo, 2020), moderators, or mediators between SRL and academic achievement (e.g., Teng and Zhang, 2018; Tian et al., 2018). In the few articles about the effect of Chinese students’ SRL on academic achievement, the vast majority of them did not integrate SRL’s components under a single rubric named “SRL” (e.g., Li et al., 2015; Liu et al., 2019; Wu et al., 2020). Given that not all self-regulated strategies can enhance academic achievement (Li et al., 2018), not treating SRL as an integrated concept is insufficiently rigorous and need to be improved. Further, empirical studies on the nature of Chinese students’ SRL, however, are lacking. In this connection, this paper attempts to explore the SRL of 15-year-old Shanghai students as well as the effect of SRL on reading literacy on the basis of PISA 2009 for Shanghai-China. The reason why we take Shanghai as an example is Shanghai’s successful performance in the Program for International Students Assessment (PISA), which has attracted attention from the Western countries since 2009 and has been named “Shanghai Shock.” As Western countries also view Shanghai students as passive learners, who often use memorization strategy that is not conducive to academic achievement, so Western countries were shocked by Shanghai students’ performance in PISA. By exploring the nature of SRL of Shanghai students, this paper attempts to unravel the concept of “Shanghai Shock” from the perspective of SRL. This paper also tries to reveal whether or not there exist heterogeneous effects of SRL on reading literacy in Shanghai-China compared with that in the Western countries. Hence, the research questions are: (1) Are Shanghai students passive learners in terms of SRL? (2) What are the effects of SRL on reading literacy in the Shanghai context?

## Literature Review

### Chinese Students in Terms of Self-Regulated Learning

Although Chinese students have been thought to be passive learners for a long time, they outperform Western students in terms of academic achievement, which is called “the paradox of Chinese learners” (Watkins and Biggs, 1996; Chan and Rao, 2009). Nowadays, “Shanghai Shock” is deemed as a vivid example of the paradox. From the perspective of SRL, the paradox of Chinese learners is that students use memorization strategy frequently but perform well in academic achievement. Many scholars tried to unravel the paradox of Chinese learners (e.g., Watkins and Biggs, 1996; Chan and Rao, 2009). Some of them held the view that scholars wrongly used the Western lens to interpret learning in the Chinese context. Unlike Western students, the memorization strategy exerts a positive influence over academic achievement in the Chinese context as memorization or rehearsal strategy in the Chinese context involves understanding (Rao and Sachs, 1999; Dahlin and Watkins, 2000; Chan and Rao, 2009). However, rehearsal learning without deep understanding was a true perception rather than a Western misperception (Morrison and Tang, 2002). Further, some empirical studies did not find a significantly positive relationship between rehearsal learning/memorization and academic achievement (e.g., Richardson et al., 2012; Lau and Ho, 2016). Liu et al. (2019) overturned the premise that Chinese learners were only good at rote memorization by drawing on 48,000 eighth-grade Chinese students. Also, the real reason why Chinese students outperform Western students is that they work hard, know the value of schooling, and are good test-takers, and the contents of some international tests are suited to the contents of Chinese curricula (Morrison, 2006). As for “Shanghai Shock,” Li et al. (2013) revisited the premise of the shock and consequently found that the stereotype that Shanghai students use memorization frequently are not true by using the PISA 2009 dataset. This finding means that the paradox is not valid for Shanghai students.

### The Effect of Self-Regulated Learning on Academic Achievement

In the Western countries, a large number of empirical studies showed a positive effect of SRL on academic achievement in junior secondary schools. For example, Fadlelmula et al. (2015) surveyed 1,019 seventh graders enrolled in public elementary schools in Ankara-Turkey and employed structural equation modeling (SEM) to explore the relationship between SRL and mathematics achievement. Their results revealed that mastery goal orientation, self-efficacy, and elaboration were significantly related to mathematics achievement. The positive effect of SRL on academic achievement was also found in senior secondary schools. Drawing on a longitudinal data, Nota et al. (2004) found that organizing and transforming exerted a positive effect on Italian, mathematics, technical subjects in high school, course grades, and examinations passed at the university. Self-consequences exerted a positive effect on students’ high school diploma grades and their intention to continue with their education after high school. Besides, León et al. (2015) surveyed 1,412 high-school students and employed SEM to explore the effects of autonomy, motivation, and self-regulated learning on mathematical achievement. Their results revealed that motivation mediated the effect of autonomy on regulation of effort, and regulation of effort could positively predict mathematical achievement. Veas et al. (2019) investigated the direct relationship between SRL and academic achievement, in particular with metacognitive strategy. By using 1,398 high-school students in Spain, their study found that metacognitive strategy exerted a direct effect over academic achievement and was also an important mediator for parental involvement’s indirect effect on academic achievement. Lenes et al. (2020) examined the effect of students’ SRL acquired in kindergarten on academic achievement in grade 1 and five by drawing on a longitudinal data for Norway, and empirically showed an importance of early SRL for later academic achievement.

Some studies showed a positive effect of SRL on academic achievement in Chinese societies. Ho (2004) used data from the first cycle of PISA to investigate the effect of SRL on academic literacy in Hong Kong. Her study revealed that control strategy and self-efficacy exerted a positive effect on mathematical, scientific, and reading literacy. In addition, instrumental motivation and memorization had a negative effect on mathematical and scientific literacy. Recently, Lau and Ho (2016) used PISA 2009 for Hong Kong to explore the effect of self-regulated reading learning on reading literacy. They found that metacognition in understanding and remembering, metacognition in summarizing, and control strategy were conducive to reading literacy. They also found that cognitive strategy appeared to be significantly related to reading literacy. In Mainland China, however, studies investigating the effectiveness of students’ SRL in basic education appeared several years ago (e.g., Liu and Chen, 2000; Gao et al., 2011), because attention has shift from effectiveness to determinants of SRL in last decade. Li et al. (2018) carried out a meta-analysis, and showed a medium relationship between SRL and academic achievement. Recently, some studies have paid more attention to SRL under the context of online learning (e.g., Xu and Zhu, 2019). Others investigated the cognitive strategy subscale of SRL. For example, Liu et al. (2019) found that combined learning strategies are positively correlated with students’ mathematical achievement by employing 48,000 eighth-grade Chinese students. Wu et al. (2020) found that students who use metacognitive strategy along with memorization or elaboration strategy achieved better in mathematics by drawing on PISA 2012 for Shanghai.

To sum up, it is clear that some research gaps still need to be filled in. First, from the perspective of SRL, the concept of “Shanghai Shock” and its relationship with “the paradox of Chinese learners” are unclear. Second, though some studies have investigated the effects of SRL on reading literacy, evidence from Mainland China is still lacking.

## Materials and Methods

### Participants

This paper used data from the OECD’s Programme for International Student Assessment (PISA) 2009 for Shanghai. Although the data is a bit outdated, so far, only PISA 2009 for Shanghai contains the SRL questionnaire. Hence, the data is very valuable for the analysis of SRL in Shanghai-China. PISA is a triennial international survey, which aims to evaluate education systems worldwide by testing 15-year-old students’ literacy, that is, cognitive achievement in applying knowledge and skills in main subjects, as well as in analyzing, reasoning, and communicating successfully in a lot of situations (OECD, 2010b). PISA 2009 for Shanghai covered 5,115 students representing about 100,000 15-year-old students in Shanghai. The observations used for analysis were 4,841. The research sample included 2,481 males (48.75%) and 2,360 females (51.25%). The mean international grade of the sample adolescents was 9.54 (*SD* = 0.63, range 7–12). 1,981 (40.92%) students were from general middle schools, 1,839 (37.99%) were from general high schools, and 1,021 (21.09%) were from vocational schools.

### Measures

#### Reading Literacy

This paper focuses on reading literacy measured by the first plausible value in reading test score. The mean of reading literacy for OECD countries was 500, and the standard deviation was 100. The mean and standard deviation of reading literacy of 15-year-old students in Shanghai was 558.26 and 78.58, respectively.

#### Self-Regulated Learning

**FIGURE 1:**
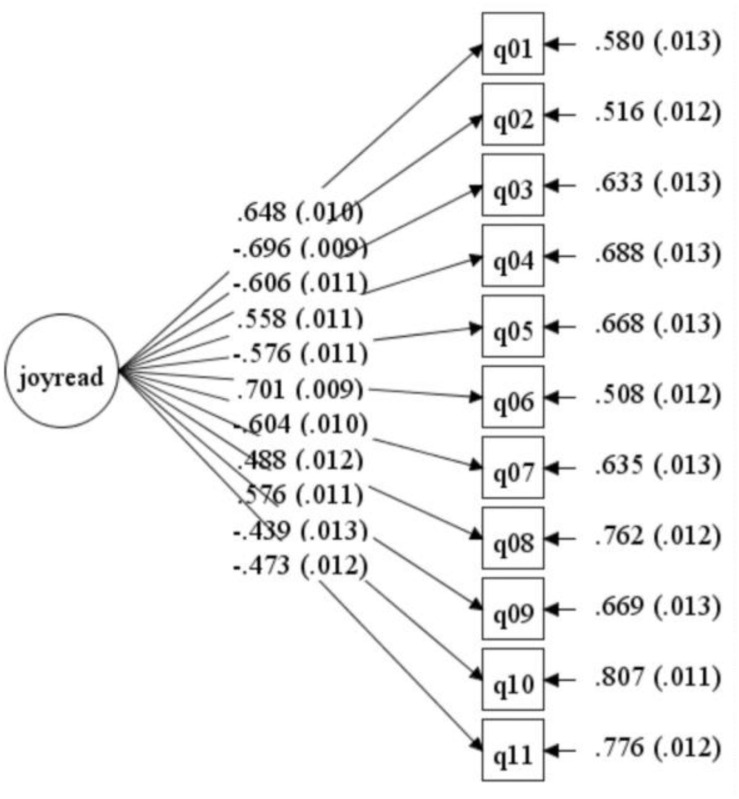
Confirmative factor analysis of enjoyment of reading.

The most used SRL construct consists of three dimensions: motivational belief, cognitive strategy, and metacognition (Pintrich and De Groot, 1990; Ramdass and Zimmerman, 2011). These three dimensions constitute the working definition of SRL in this paper, which was operationalized via self-regulated reading learning and measured with PISA questionnaire. First, although OECD uses a different terminology, the concept of reading enjoyment is actually one of the motivational belief (OECD, 2010a). Hence, motivational belief in this paper is measured by enjoyment of reading. See Table 1 for the details of the items. Students were asked to answer on a four-point Likert scale ranging from 1 (strongly disagree) to 4 (strongly agree). All items that are negatively phrased (items 1, 4, 6, 8, 9) were reverse scored; hence, positive score indicates a higher level enjoyment of reading. Confirmative factor analysis (CFA) was carried out by using Mplus 8.3 (see Figure 1) and affirmed the satisfactory construct validity of the enjoyment of reading (χ^2^ = 1,365.642, df = 44, RMSEA = 0.079, TLI = 0.910, CFI = 0.887). The Cronbach’s α coefficients for enjoyment of reading subscales (0.84) indicated good reliability.

**TABLE 1:** Items for the variables of self-regulated learning (SRL) measured in this study.

SRL subscales	Items
Enjoyment of reading	st24q01: I read only if I have to.
	st24q02: Reading is one of my favorite hobbies.
	st24q03: I like talking about books with other people.
	st24104: I find it hard to finish books.
	st24q05: I feel happy if I receive a book as a present.
	st24q06: For me, reading is a waste of time.
	st24q07: I enjoy going to a bookstore or a library.
	st24q08: I read only to get information that I need.
	st24q09: I cannot sit still and read for more than a few minutes.
	st24q10: I like to express my opinions about books I have read.
	st24q11: I like to exchange books with my friends.
Memorization strategy	st27q01: When I study, I try to memorize everything that is covered in the text.
	st27q03: When I study, I try to memorize as many details as possible.
	st27q05: When I study, I read the text so many times that I can recite it.
	st27q07: When I study, I read the text over and over again.
Elaboration strategy	st27q04: When I study, I try to relate new information to prior knowledge acquired in other subjects.
	st27q08: When I study, I figure out how the information might be useful outside school.
	st27q10: When I study, I try to understand the material better by relating it to my own experiences.
	st27q12: When I study, I figure out how the text information fits in with what happens in real life.
Control strategy	st27q02: When I study, I start by figuring out what exactly I need to learn.
	st27q06: When I study, I check if I understand what I have read.
	st27q09: When I study, I try to figure out which concepts I still have not really understood.
	st27q11: When I study, I make sure that I remember the most important points in the text.
	st27q13: When I study and I do not understand something, I look for additional information to clarify this.

**FIGURE 2:**
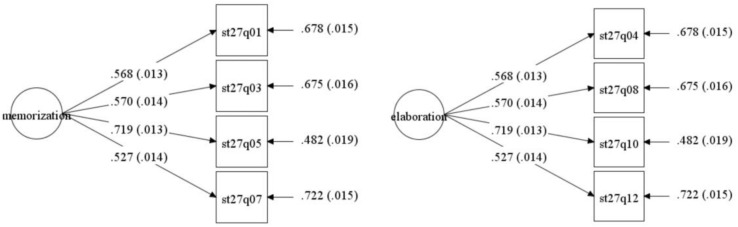
Confirmative factor analysis of cognitive strategy.

Second, cognitive strategy consists of two scales: memorization and elaboration. Memorization strategy is to store information as it is, without much further processing. Elaboration strategy requires students to use the knowledge acquired to some degree (OECD, 2010a). Students were asked to answer on a four-point Likert scale ranging from 1 (almost never) to 4 (almost always). Positive scores on a given learning strategy index indicate greater use of that learning strategy. Confirmative factor analysis (CFA) of the present sample (see Figure 2) affirmed the satisfactory construct validity of the memorization (χ^2^ = 59.254, df = 2, RMSEA = 0.077, TLI = 0.943, CFI = 0.981) and elaboration strategies scale (χ^2^ = 59.254, df = 2, RMSEA = 0.077, TLI = 0.943, CFI = 0.981). The Cronbach’s α coefficients for memorization (0.64) and elaboration strategies subscales (0.71) indicated good reliability.

**FIGURE 3:**
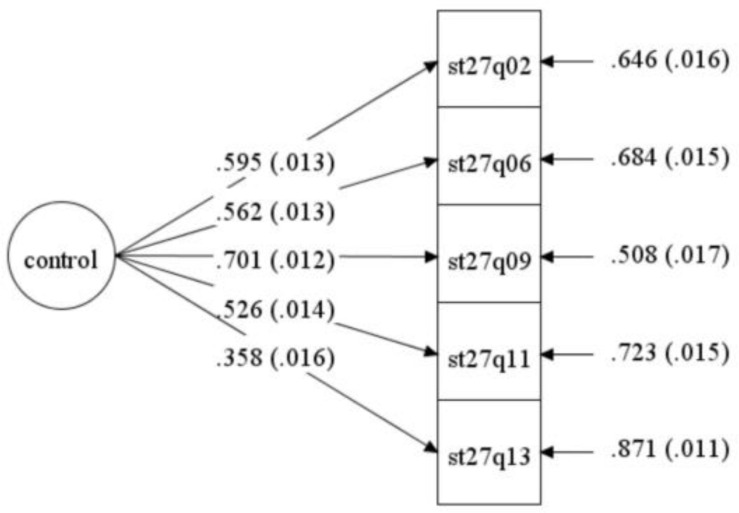
Confirmative factor analysis of control strategy.

Third, high-level cognition was labeled as “metacognition” by Flavell in the mid-1970s, which includes (Baker, 2010): (1) planning, checking, evaluating, remediating, and revising strategies; (2) knowledge about ourselves as learners, about aspects of the task, and about strategy use. As such, metacognition consists of two scales in this paper: (1) control strategy; (2) metacognition in understanding and remembering, and metacognition in summarizing. Control strategy is one component of metacognition that involves planning, monitoring, and regulation, which is essential for SRL (OECD, 2005). See Table 1 for the details of the items. Students were asked to answer on a four-point Likert scale ranging from 1 (almost never) to 4 (almost always). Positive score on control strategy index indicates frequent use of control strategy. Confirmative factor analysis (CFA) of the present sample (see Figure 3) affirmed the satisfactory construct validity of the control strategy scale (χ^2^ = 156.399, df = 5, RMSEA = 0.079, TLI = 0.914, CFI = 0.957). The Cronbach’s α coefficients for control strategy subscales (0.71) indicated good reliability.

Metacognition in understanding and remembering and summarizing assesses the extent to which a student is aware of what strategies are appropriate to understand and remember and summarize information (OECD, 2010a). Metacognition reflects a student’s awareness of effective strategies. The indices of metacognition in understanding and remembering and summarizing are computed as follows: To determine ordering for each task such as summarizing information, students were asked to rate the usefulness of each strategy, and then the proportion of the total number of expert pairwise relations that were consistent with the student ordering was computed. The final scores that each student got for each task was a number that ranged from 0 to1 (OECD, 2012).

#### Control Variables

The control variables consist of covariates at student level and school level. Regarding control variables at student level, the index of reading diversity was 0.44, while the index of online reading was −0.35. The low index of online reading indicated that Shanghai 15-year-old students had less opportunity to read online than their counterparts in OECD countries. Girls accounted for 51.25%. The majority of 15-year-old students were from general middle schools (40.92%), followed by general high schools (37.99%), upper secondary vocational schools (20.41%), and 6/7-year secondary vocational schools (0.68%). Students born in Shanghai accounted for 84.47% of the total, followed by 14.56% born in other provinces, 0.23% born in Hong Kong, Macau, or Taiwan, and 0.74% born in foreign countries. Family characteristics included socioeconomic status and family structure. Socioeconomic status was measured by the variable “SES,” whose mean (−0.48) was lower than the OECD average (0). Students from two-parent family, single-parent family, and other family structure accounted for 86.59%, 10.60%, and 2.81%, respectively. Control variables at school level included school type, school resources, school size, student–teacher ratio, teacher quality, teacher participation, school average SES, and school climate. Private schools accounted for 10.23% of all schools. The means of shortage of teaching staff and quality of educational resources were 0.57 and 0.15, respectively. The school size was 1,660 and the student–teacher ratio average was about 14. About 14.97% of teachers had teaching certification. Teacher participation in decision making (0.86) was higher than the OECD average. The school average SES was −0.48. School climate measured by student factors such as student absenteeism was 0.10.

### Analysis

A random intercept model of multilevel linear regression was used as the statistical model to investigate the effect of self-regulated reading learning on reading literacy. The statistical package used is STATA 14.0. First, a null model was developed to illustrate between- and within-school variance of reading literacy.

Null Model:

$$\begin{array}{l} y_{\text{ij}}=\beta_{0j}+\epsilon_{\text{ij}}\\ \beta_{0j}=\gamma_{00}+\mu_{0j} \end{array}$$

Second, baseline models with SRL subscales were being investigated in turn. Baseline models demonstrate the correlations between SRL and reading literacy, while not controlling covariates.

Baseline Model:

$$\begin{array}{l} y_{\text{ij}}=\beta_{0j}+\beta_{1j}s_{\text{ij}}+\epsilon_{\text{ij}}\\ \beta_{0j}=\gamma_{00}+\mu_{0j};\beta_{1j}=\gamma_{10} \end{array}$$

Third, an extension model controlling for the covariates at student level on the basis of baseline model was developed, and a final model controlling for the covariates at both student and school level was developed. These models were developed to netting out covariates’ confounding effects.

Extension Model:

$$\begin{array}{l} y_{\text{ij}}=\beta_{0j}+\beta_{1j}s_{\text{ij}}+\sum_{k=2}^{K}\beta_{\text{kj}}x_{k,\mathrm{ij}}+\epsilon_{\text{ij}}\\ \beta_{0j}=\gamma_{00}+\mu_{0j};\beta_{\text{kj}}=\gamma_{\text{k}0},k=1,\ldots \ldots \text{K} \end{array}$$

Final Model:

$$\begin{array}{l} y_{\text{ij}}=\beta_{0j}+\beta_{1j}s_{\text{ij}}+\sum_{k=2}^{K}\beta_{\text{kj}}x_{k,\mathrm{ij}}+\epsilon_{\text{ij}}\\ \beta_{0j}=\gamma_{00}+\sum_{n=1}^{N}\gamma_{0n}z_{n,j}+\mu_{0j};\beta_{\text{kj}}=\gamma_{\mathrm{k0}},k=1,\ldots \ldots \text{K} \end{array}$$

In the above equations, β_*0j*_ is the intercept for school *j*; *y*_*ij*_ is the reading literacy for student *i* in school *j*; *s*_*ij*_ is the self-regulated reading learning for student *i* in school *j*; *x*_*k,ij*_ is the student’s characteristics for student *i* in school *j*; *z*_*n,j*_ is the school’s characteristics for school *j*; μ_*0j*_ is the stochastic disturbance for school *j*; and ϵ_*ij*_ is the stochastic disturbance for student *i* in school *j*.

## Results

### Self-Regulated Learning of Shanghai Students

**FIGURE 4:**
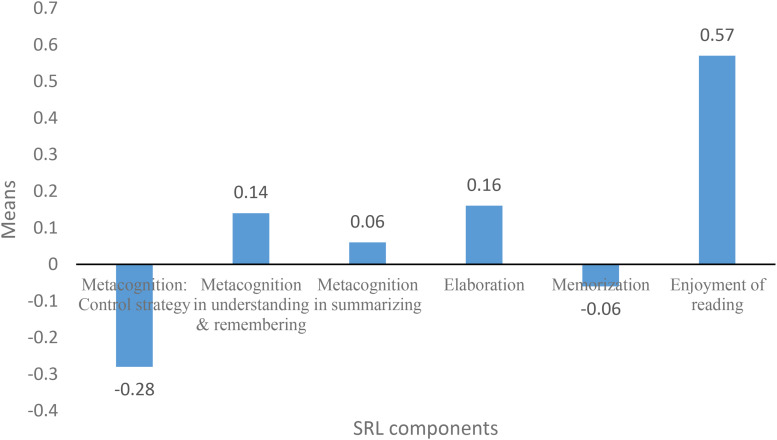
The means of components of SRL.

Chinese students are stereotyped as passive rather than proactive learners, so the means of Shanghai students’ self-regulated reading learning, as measured by cognitive strategy, metacognition, and enjoyment of reading, are expected to be lower than that of their counterparts in the Western countries. This is because of the emphasis on confirmation and respect for authority in Confucian Heritage Culture (CHC), which encourages passive learning without engaging students in learning proactively (Lau and Ho, 2016). As expected, the mean of the control strategy (−0.28) used by 15-year-old students in Shanghai was below the mean of their counterparts in OECD countries (see Figure 4). Regarding the means of other metacognition subscales, cognitive strategies, and enjoyment of reading, however, Shanghai 15-year-old students are not as expected; they are well aware of effective strategies, use elaboration strategy frequently, while seldom using memorization strategy, as well as they enjoy reading. Specifically, Shanghai 15-year-old students scored far above the students in OECD countries in terms of metacognition in understanding and remembering and summarizing, as the means of metacognition in understanding and remembering and summarizing were 0.14 and 0.06, respectively (see Figure 4). Similarly, with regard to cognitive strategy, the use of elaboration strategy (0.16) among Shanghai 15-year-old students was higher than that of their counterparts in OECD countries, and the use of memorization strategy (−0.06) was lower than that of their counterparts in OECD countries (see Figure 4). Moreover, the enjoyment of reading of Shanghai 15-year-old students was 0.57, which scored far above the students in OECD countries (see Figure 4).

As mentioned before, there exists the paradox, namely, Chinese learners are thought to be passive learners but perform well, which has confused scholars for a long time. However, in the Shanghai case, the paradox is not valid since Shanghai students are not passive learners from the perspective of SRL. The data above revealed that though Shanghai students seldom used control strategy to regulate cognitive process, they were well aware of effective strategies to understand, remember, and summarize information, and used elaboration strategy instead of memorization strategy frequently. In other words, Shanghai students could not be fully viewed as passive learners as reflected in their SRL compared with students in the Western countries.

**FIGURE 5:**
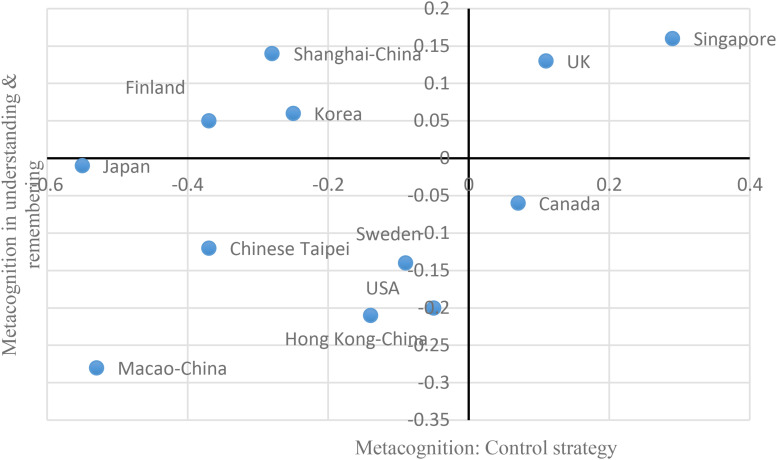
Control strategy and metacognition in understanding and remembering.

**FIGURE 6:**
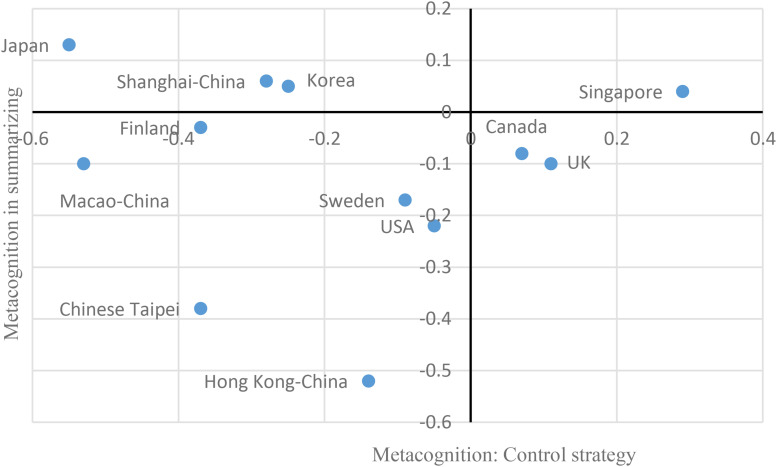
Control strategy and metacognition in summarizing.

Shanghai shares some commonalities with Chinese societies. In the context of CHC as well as “teaching to test scores,” students may not be motivated to use control strategy to regulate the learning process. This happened in Shanghai, Taipei, Hong Kong, and Macao, which located in the left half of graphs (see Figures 5, 6). However, treating students among Chinese societies as a single and homogeneous group may misrepresent the diversity (Morrison, 2006). Shanghai has its own uniqueness. Once Shanghai students began their learning processes whether by themselves or not, they were aware of effective strategies to understand, remember, and summarize information (see the top half of Figures 5, 6). In addition to awareness, some cognitive strategies mastered by Shanghai students could be used in the learning process, such as elaboration strategy. Students in the other Chinese societies—Taipei, Hong Kong, and Macao, however, were poorly aware of effective strategies (see the down half of Figures 5, 6).

To sum up, on the one hand, students in Shanghai and the other Chinese societies (Taipei, Hong Kong, and Macao) seldom used control strategy. On the other hand, regarding awareness of effective strategies, Shanghai students outperformed students in the other Chinese societies that were characterized by poor awareness of effective strategies. Actually, Shanghai students were similar to those in Japan, Korea, and Finland because student in these countries were characterized by low use of control strategy but excellent awareness of effective strategies and excellent performance.

### Correlation Between Self-Regulated Learning and Reading Literacy

In the pairwise correlations among SRL components, the reading literacy was analyzed by Pearson correlation (see Table 2). With regard to correlation between cognitive strategy and metacognition, this paper found that either elaboration strategy (*r* = 0.608, *p* < 0.01) or memorization strategy (*r* = 0.536, *p* < 0.01) was highly positively associated with control strategy, and that memorization strategy was not related to metacognition in summarizing. In addition, enjoyment of reading was positively associated with cognitive strategy and metacognition.

**TABLE 2:** Pearson correlation coefficients.

	*n*	*M*	*SD*	1	2	3	4	5	6	7
(1) Elaboration	4841	0.16	0.81	–						
(2) Memorization	4841	−0.06	0.79	0.397***	–					
(3) Metacognition: control	4841	−0.27	0.81	0.608***	0.536***	–				
(4) Metacognition: summarizing	4841	0.08	0.89	0.098***	0.017	0.159***	–			
(5) Metacognition: understanding and remembering	4841	0.15	0.96	0.070***	0.043***	0.150***	0.394***	–		
(6) Enjoy of reading	4841	0.58	0.69	0.296***	0.306***	0.365***	0.168***	0.171***	–	
(7) Reading literacy	4841	558.26	78.58	0.149***	0.054***	0.276***	0.374***	0.331***	0.358***	–

*p < 0.1, **p < 0.05, ***p < 0.01.

Within cognitive strategy, a medium positive correlation (*r* = 0.397, *p* < 0.01) was found between elaboration and memorization strategy. Among the three dimensions of metacognition, a small to medium positive pairwise correlation was found. Specifically, the correlation between control strategy and metacognition in summarizing, metacognition in understanding and remembering were 0.159 (*p* < 0.01) and 0.15 (*p* < 0.01) respectively, and the correlation between metacognition in summarizing and metacognition in understanding and remembering was 0.394 (*p* < 0.01). In addition, all components of SRL were positively associated with reading literacy.

### Results From Multilevel Linear Regression

Table 3 shows the effect of SRL on reading literacy in Shanghai-China by using multilevel linear regression. In the null model, the between-school variance accounted for 43.64% of the total variance and within-school variance accounted for 56.36% of the total variance.

**TABLE 3:** The effects of SRL on reading literacy.

	Model 1	Model 2	Model 3	Model 4	Model 5
Fixed effects					
Intercept					
Elaboration	6.659***			3.138***	2.915***
	(1.189)			(1.066)	(1.058)
Memorization	0.161			−5.371***	−5.197***
	(1.391)			(1.149)	(1.145)
Metacognition					
Summarizing		13.634***		11.950***	11.972***
		(1.148)		(1.067)	(1.064)
Understanding and remembering		11.288***		9.585***	9.529***
		(0.999)		(0.993)	(0.991)
Control		11.014***		6.189***	6.017***
		(1.344)		(1.275)	(1.261)
Enjoyment of reading			27.114***	19.654***	19.662***
			(1.624)	(1.535)	(1.541)
Covariates at student level				YES	YES
Covariates at school level					YES
Random effects					
Between-school variance	2,636.788	2,007.340	2,311.667	827.149	246.464
Within-school variance	3,474.730	3,075.050	3,180.378	2,783.088	2,783.417

Total N = 4,841. Robust cluster standard errors in parentheses; *p < 0.1, **p < 0.05, ***p < 0.01. Given the multicollinearity between cognitive strategy (elaboration or memorization) and control strategy, the coefficient of control strategy is from a model with metacognition variables together with enjoyment of reading. The other coefficients are from a model with elaboration and memorization strategies instead of control strategy, together with metacognition in understanding and remembering, in summarizing and enjoyment of reading. If interested in coefficients of covariates, please contact the author.

In the first step, the null model was expanded with self-regulated reading learning to develop the baseline model (i.e., Models 1, 2, 3). As shown in Table 3, the percentages of between-school variance, within-school variance, and total variance explained were 2.72, 0.73, and 1.60%, respectively, in Model 1 with cognitive strategy, 25.94, 12.15, and 18.17%, respectively, in Model 2 with metacognition, and 14.71, 9.14, and 11.57%, respectively in Model 3 with enjoyment of reading. All SRL components but memorization strategy had a significantly positive effect on students’ reading literacy without controlling for any student or school variables. More specifically, an increase in one unit on elaboration strategy was associated with a reading literacy of 6.659 points in Model 1. In Model 2, an increase in one unit on control strategy was associated with a reading literacy of 11.014 points, an increase in one unit on metacognition in summarizing was associated with a reading literacy of 13.634 points, and an increase in one unit on metacognition in understanding and remembering was associated with a reading literacy of 11.288 points. An increase in one unit on enjoyment of reading was associated with a reading literacy of 27.114 points in Model 3.

Second, the baseline model was expanded further with control variables at student level to develop the extension model (i.e., Model 4). The percentages of between-school variance, within-school variance, and total variance explained were 69.48, 20.49, and 41.48%, respectively, in Model 4. This time, originally significant SRL components still had a significantly positive effect on students’ reading literacy, with the coefficients becoming smaller than the corresponding coefficient in Models 1, 2, 3. However, the memorization strategy had a significantly negative effect on students’ reading literacy.

In the last step, covariates at school level were controlled on the basis of the extension model (i.e., Model 5). The percentages of between-school variance, within-school variance, and total variance explained were 90.91, 20.48, and 51.22%, respectively in Model 5. It should be noted that all SRL components, but memorization, still had a significantly positive effect on students’ reading literacy, and the magnitudes were almost the same as the corresponding coefficients in Model 4. The memorization strategy still had a significantly negative effect on students’ reading literacy.

## Discussion

As now we understand things, the stereotyped impression of Chinese learners may not remain valid for today’s Shanghai students as they seldom use memorization strategy, while using elaboration strategy frequently during the learning process. This phenomenon may be explained by a recent reform in Shanghai. OECD (2011) related the successful performance of Shanghai to curriculum reform. National curriculum reform in 2001 called for changes in the way that repetitive rote learning moved toward the ability to acquire new knowledge and to analyze and solve problems, through learning activities such as student participation, real-life experiences, communications, and teamwork. Indeed, the aim of pedagogy reform was to return class time to students (Cheng, 2011). A teacher-centered teaching practice was replaced by student-centered teaching practice, such as individual project work (Tan and Chua, 2015). Under the Shanghai curriculum reform, Shanghai teachers may not only feed students “fish” but also teach students how to “fish” (i.e., awareness of effective strategies and use of high-order cognitive strategies).

Based on the findings from the multilevel linear regression, memorization strategy was not found to be conducive to reading literacy for Shanghai students who were stereotyped as rote learners, which was corroborated by previous evidence from Western countries about the effect of self-regulated learning on academic achievement (e.g., Nota et al., 2004; Fadlelmula et al., 2015; León et al., 2015). Moreover, these findings are consistent with evidence from Chinese literatures of components of SRL. Enjoyment of reading is a strong predictor of academic achievement (Lee, 2014). In the aspect of cognitive strategy, memorization strategy that is thought to be a surface cognitive strategy has a negative effect on students’ academic achievement, while elaboration strategy that is thought to be a deep cognitive strategy could help students process information more deeply (Liu et al., 2019). In the aspect of metacognition, students who exhibit higher levels of metacognitive knowledge about effective strategies are more successful in academic achievement (Wu et al., 2020). Control strategy, which mobilizes metacognitive knowledge strategically and monitor cognitive strategies (Wu, 2018) were positively correlated with students’ academic achievement (Liu et al., 2019). To sum up, the results imply that teaching students about different cognitive strategies, helping students to foster awareness of strategies, and controlling the learning process may be important in improving academic achievement in Shanghai.

Combined with the discovery of false premise of “Shanghai Shock,” 15-year-old students in Shanghai could academically succeed like Western students because they use high-order cognitive strategies such as elaboration strategy, and they also enjoy reading. On the other hand, by comparing Shanghai with other countries or Chinese societies, this paper found the uniqueness of Shanghai students in terms of awareness of effective strategies, that is, they have high metacognition in understanding, remembering, and summarizing. Hence, the secret why Shanghai students could outperform Western students and rank highest on the academic achievement may be the high awareness of effective strategies, but this hypothesis needs to be addressed in future studies. Although Shanghai students foster awareness of strategies, mobilize cognitive strategy, and enjoy reading through recent reform, it is still not enough to sustain a successful performance. Rather, students must use control strategy to regulate their learning processes (Pintrich and De Groot, 1990). As such, Shanghai 15-year-old students have not fostered the habit of controlling their own learning processes. Hence, Shanghai could learn from the successful experience in Singapore in terms of the high use of control strategy and excellent awareness of effective strategies.

## Conclusion and Limitations

By using PISA2009 for Shanghai, this paper examined the nature of Shanghai students’ SRL, as measured by elaboration strategy, memorization strategy, metacognition in understanding and remembering, metacognition in summarizing, control strategy, and enjoyment of reading, and then investigated the effect of SRL on reading literacy. In the aspect of SRL’s nature, this paper found that 15-year-old students in Shanghai were well aware of strategies to understand, remember, and summarize information, while they seldom used control strategy during learning process. With regard to the cognitive strategy component of SRL, Shanghai students used elaboration strategy frequently, while they seldom used memorization strategy. Moreover, they enjoyed reading very much. In the aspect of SRL’s consequence for reading literacy, the results also revealed that among the SRL components, metacognition in understanding and remembering, metacognition in summarizing, control strategy, elaboration strategy, and enjoyment of reading were conducive to students’ reading literacy, whereas memorization strategy exerted a significantly negative effect on reading literacy.

The theoretical or research contribution of this paper is twofold. On one hand, it clarifies the nature of today’s Shanghai students, that is, they are not passive learners who use memorization strategy frequently. On the other hand, this paper shows a similar pattern in the effects of components of SRL as those in the Western countries. In particular, the effect of memorization strategy on academic achievement is negative in Shanghai as well as in the Western countries. The two aspects mentioned above help us to gain a better understanding of Shanghai students. It should be noted that this paper is not without limitations. First, this paper did not investigate the effect of SRL on mathematical or scientific literacy because of data limitation. The effect of SRL on reading literacy may not be the same as the effect on mathematical or scientific literacy; future researches could fill the gap through investigating the effectiveness on other subjects. Second, results from this paper just illustrated self-regulated learning of Shanghai students and thus should not be simply generalized to Chinese Mainland students. Third, though this paper brings up the hypotheses that metacognition or awareness of effective strategies may be the key factors contributing to “Shanghai Shock,” it does not address the hypotheses. Hence, further studies could be conducted to explore whether and, if so, how, metacognition or awareness of effective strategies is the key explanatory factor for “Shanghai Shock.”

## Data Availability Statement

Publicly available datasets were analyzed in this study. This data can be found here: http://www.oecd.org/pisa/data/pisa2009database-downloadabledata.htm.

## Author Contributions

XQ designed this study, analyzed the data, and wrote this article.

## Conflict of Interest

The author declares that the research was conducted in the absence of any commercial or financial relationships that could be construed as a potential conflict of interest.
